# Effects of mind-body exercise on cardiopulmonary function, blood pressure, and quality of life in CHD patients: A protocol for systematic review and meta-analysis

**DOI:** 10.1097/MD.0000000000025042

**Published:** 2021-03-05

**Authors:** Fei-xing Duan, Qi Wu, Yi-fan Zuo, Hui-xin Yang, Fei Dai, Lin Wang

**Affiliations:** a Department of Physical Education, Wuhan University of Technology, Wuhan; b Traditional Sport Institute, Harbin Sport University, Harbin; c Institute of Physical Education, Wuchang Institute of Technology, Wuhan, China.

**Keywords:** blood pressure, cardiopulmonary function, CHD patients, meta-analysis, mind-body exercise, quality of Life, systematic review

## Abstract

**Background:**

Coronary heart disease (CHD) is one of the highest mortality diseases in the world, which seriously threatens human health and quality of life (QOL). The purpose of this study is to systematically analyze the effects of mind-body exercise on cardiopulmonary function, blood pressure and QOL in CHD patients, and to provide scientific evidence-based exercise prescription for patients with coronary heart disease.

**Methods:**

This research review will include the following electronic databases from its establishment to December 2020: PubMed, EMBASE, Web of Science, Cochrane Library, the Chinese National Knowledge Infrastructure, the Chinese Science and Technology Periodical Database, and Wanfang. Objective to search randomized controlled trials (RCTs) about the effects of mind-body exercise on cardiopulmonary function, blood pressure and QOL in patients with coronary heart disease.

**Conclusion:**

This systematic review and meta-analysis will provide strong evidence for the efficacy and safety of mind-body exercise in patients with coronary heart disease.

**Systematic review registration:**

INPLASY202120016.

**Ethics and dissemination:**

Ethical approval will not be necessary since this systematic review and meta-analysis will not contain any private information of participants or violate their human rights.

## 1 Introduction

Coronary heart disease (CHD) has become one of the main causes of death in the world. Nearly every 25 seconds, an American will have a coronary event related to CHD, and every minute someone will die from one.^[1]^ According to data from the World Health Organization (WHO), around 80% of the risk factors for CHD can be influenced by the patient's behavior; this applies above all to lack of exercise, poor nutrition, overweight, and chronic subjective stress.^[2–3]^ In fact, major risk factors for CHD, including physical inactivity, overweight, hypertension (HTN), and elevated serum lipids, remain a major problems for most Americans.^[1]^

In recent years, the treatment of coronary heart disease in addition to conventional treatment also includes some nonpharmacological alternative therapy. Regular physical activity in the form of exercise has been used as an alternative therapy for many cardiovascular diseases, particularly CHD.^[4]^ It is well documented that exercise prevents and even reverses CHD.^[5]^ The U.S. Department of Health and Human Services (2008) formulated a recommendation on health promotion and disease prevention, in which reducing cardiovascular deaths and encouraging exercise are the 2 main goals. Therefore, health care providers are encouraged to reduce the risk of CHD by recommending appropriate aerobic exercises to patients to promote a healthy lifestyle. Some studies have shown that exercise training can effectively strengthen the aerobic metabolism of patients with coronary heart disease, which is conducive to the regulation of blood pressure, blood glucose, and blood lipid of patients.^[6]^ In addition, it can also improve the cardiopulmonary function of patients with myocardial infarction, reduce the incidence of acute coronary artery events and readmission rate of patients,^[7]^ and then improve the quality of life (QOL) of patients.^[8–9]^ Ornish et al^[10]^ showed that the risk of sudden death in acute myocardial infarction patients receiving cardiac rehabilitation decreased by 45% within 1 year. As a way to promote physical and mental health, mind-body exercise has attracted the attention of many scholars in recent years. Mind-body exercise focuses on mind, body, psychology, and behavior, which is an effective combination of breathing and physical activities,^[11–12]^ emphasizing the advantages of mind-body exercise at the same time.

## 2 Objectives

Objective to systematically evaluate the effects of mind-body exercise on cardiopulmonary function, blood pressure, and QOL in patients with coronary heart disease (CHD). We expect to provide reference for mind-body exercise treatment of coronary heart disease.

## 3 Methods

### 3.1 Study registration

This review protocol is registered in the International Platform of Registered Systematic Review and Meta-analysis Protocols, registration number INPLASY202120016. This systematic review protocol will be conducted and reported strictly according to Preferred Reporting Items for Systematic Reviews and Meta-Analyses (PRISMA)^[13]^ statement guidelines.

### 3.2 Inclusion and exclusion criteria for study selection

#### 3.2.1 Inclusion criteria

The inclusion criteria were randomized controlled trials (RCTs), with mind-body exercise as the main form of intervention. The language of literature only includes Chinese or English.

#### 3.2.2 Exclusion criteria

1. Repeated publication;
2. Animal experiment;
3. Incomplete literature;
4. Nonrandomized controlled trials;
5. Systematic review and meta-analysis.

### 3.3 Types of participants

#### 3.3.1 Inclusion criteria

1. New York Heart Association (NYHA) classification I–III;
2. 40 years old ≤ age < 70 years old;
3. no hemodynamic disorder;
4. good compliance.

#### 3.3.2 Exclusion criteria

1. Patients with severe cardiopulmonary insufficiency and severe diseases;
2. patients with severe limb and joint diseases affecting sports rehabilitation training;
3. patients with severe infectious diseases and malignant tumors;
4. patients with uncontrolled severe complications.

### 3.4 Interventions and controls

The intervention methods of the experimental group were mind-body exercise, including Tai Chi, yoga, Qigong, meditative movement, etc. The control group included usual care, no physical activity, and no-intervention control group and other different types.

### 3.5 Type of outcome measures

#### 3.5.1 Primary outcomes

The indexes of cardiopulmonary function were maximal oxygen uptake, maximal heart rate, and left ventricular ejection fraction.

#### 3.5.2 Secondary outcomes

The following were defined as secondary outcomes:

1. Atherosclerosis;
2. Systolic and diastolic blood pressure;
3. QOL .

### 3.6 Search strategy

This research review will include the following electronic databases from its establishment to December 2020: PubMed, EMBASE, Web of Science, Cochrane Library, the Chinese National Knowledge Infrastructure, the Chinese Science and Technology Periodical Database, and Wanfang. The search terms will include “mind-body exercise,” and “yoga” or “taichi” or “tai ji quan” or “Taiji boxing” or “tai chi chuan” “qigong” or “health qigong” or “meditation,” with CHD terms including “coronary heart disease” or “CHD.”

### 3.7 Data collection and analysis

#### 3.7.1 Studies selection

**Figure 1:**
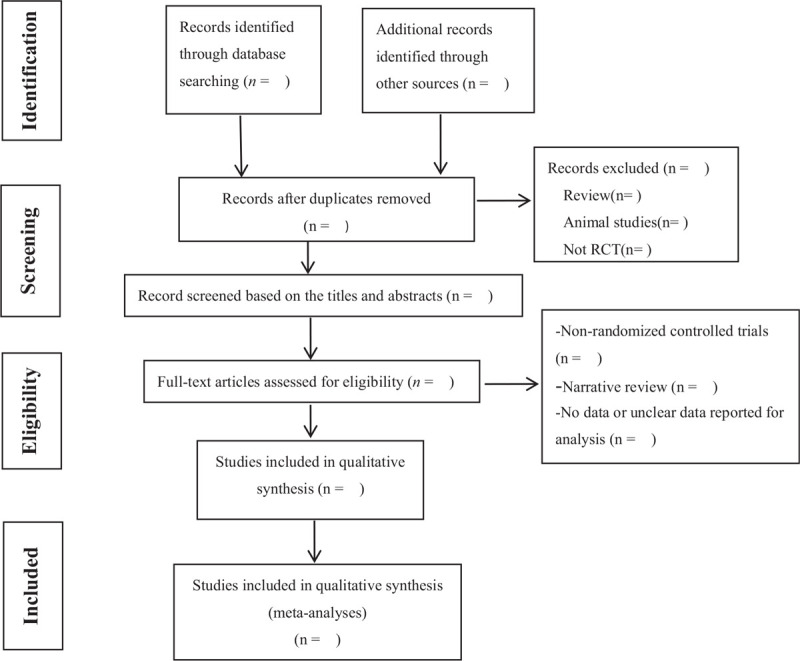
Flow diagram of study selection.

First, the 2 reviewers (Duan and Wang) will independently check the title and abstract of the search results and preliminarily screen the articles. After reading the full text of the preliminary selected articles, 2 independent reviewers selected eligible studies according to our predetermined inclusion criteria. Finally, the article selected by 2 independent commentators is integrated after putting forward repetitive parts. When 2 independent reviewers disagree, the third reviewer(Yang) will make the decision. The details of selection process will be displayed in the PRISMA flow chart^[13]^ (Fig. 1).

#### 3.7.2 Assessment of risk of bias

We will generate a funnel chart to evaluate the reporting bias. For continuous variables, Egger test will be used to test the asymmetry of funnel plot. It is generally believed that asymmetric funnel plot will present publication bias, which is one of the reporting bias, but it also means that there may be other reasons, such as methodological quality differences or real heterogeneity of intervention effect. We will analyze the possible reasons and give a reasonable explanation for the asymmetric funnel diagram.

#### 3.7.3 Measure of treatment effect

The risk ratio (CIS) of 95% confidence interval was used. Odds ratio (OR) and relative risk (RR) are commonly used for dichotomy outcome data. For continuous variables, the mean or standard deviation of 95% CI is calculated.

#### 3.7.4 Dealing with missing data

The accuracy of the data may affect the results of the study, and even lead to different conclusions. Therefore, we will contact the corresponding authors to supplement the research results with insufficient or missing data. If the corresponding author cannot be contacted, the data will be processed separately, and the potential impact of incomplete data on the results will be analyzed and reported.

#### 3.7.5 Appraisal of study quality

Two independent reviewers (Duan and Wang) will use the Modified Physical Therapy Evidence Database (PEDro) scale^[14]^ to evaluate the methodological quality of each eligible study. The assessment includes the following 11 items: Item1 = explicit inclusion criteria; Item2 = randomization; Item3 = allocation concealment; Item4 = Similar at baseline; Item5 = Subjects blinded; Item6 = Therapists blinded; Item7 = Assessors blinded; Item8 = more than 85% retention; Item9 = Intention-totreat analysis; Item10 = Between-group comparisons; Item11 = Point measures, and variability data. The score of each item is 1 (clearly described in the article), or 0 (absent or unclear). According to different total scores, each study will be divided into 3 quality levels: high (9–11), moderate (5–8), and low (0–4). Any disagreement will be resolved through discussion and negotiation with a third experienced reviewer (Yang).

#### 3.7.6 Assessment of heterogeneity

The heterogeneity assessment will be conducted by the Review Manager (v.5.3.5). According to the Cochrane Handbook, Chi-Squared test and *I*^2^ value were used to evaluate the heterogeneity. *I*^2^ values of 25%, 50%, and 75% are considered as low, moderate, and high heterogeneity, respectively.^[15]^

#### 3.7.7 Assessment of reporting biases

If there are enough studies available, we will evaluate the funnel plot to report bias.

#### 3.7.8 Data synthesis

The Review Manager (v.5.3.5) software will be used for quantitative data analysis, including mapping overall forest plot, heterogeneity analysis, regression analysis, and subgroup analysis. If the *I*^2^ value is less than 50%, it means that the relative heterogeneity is small, and the fixed effect model should be used. Otherwise, the random effect model will be used.

#### 3.7.9 Subgroup analysis

Considering the possibility of high heterogeneity, we will conduct subgroup analysis to explain the potential causes of heterogeneity when necessary. First of all, the analysis will be based on the age, gender, course of disease of patients with coronary heart disease. In addition, according to the different intensity, time and duration in the process of mind-body exercise intervention.

#### 3.7.10 Sensitivity analysis

Sensitivity analysis will be used to assess the robustness of the results. After data synthesis, we will exclude the combined studies one by one for sensitivity analysis to observe whether the comprehensive results have significant changes. If there are significant changes indicating that the exclusion study has a significant impact on the results, it should be reevaluated. On the contrary, the comprehensive data results are reliable. Chi-Squared test and *I*^2^ value will be used to quantify statistical heterogeneity.

#### 3.7.11 Quality of evidence

An internationally recognized scoring system will be used to assess the quality of our evidence. The evidence evaluation of all results will be summarized by the suggested assessment, development and assessment (GRADE) method. The level of evidence will be divided into high, moderate, low, and very low quality.

## 4 Discussion

Patients with CHD often due to coronary artery disease and lead to the corresponding myocardial blood supply, oxygen shortage, myocardial asynchronous contraction, cardiac stroke volume and circulating blood volume decreased, and then affect the cardiopulmonary function of patients. Previous studies have shown that mind-body exercise can increase coronary blood flow, improve myocardial ischemia and hypoxia, reduce cardiovascular risk factors,^[16]^ and then reduce acute cardiovascular event mortality and all-cause mortality.^[17]^ Some studies have also shown that cardiovascular disease can lead to autonomic nervous regulation function damage, mainly manifested as chronotropic insufficiency of the heart. Heart rate and blood pressure may increase during the attack.^[18]^ Heart rate and blood pressure are effective indicators reflecting the autonomic nervous regulation function of the heart. Long term mind-body exercise can also enhance the ability of cardiac autonomic nerve regulation, reduce the sympathetic nerve activity of patients with coronary heart disease, improve the tension of vagus nerve, and keep the blood pressure and heart rate in the normal range.

In recent years, various mind-body exercise intervention methods have been increasingly used in the treatment of chronic diseases. A large number of studies have explored the effects of mind-body exercise on the cardiopulmonary function, blood pressure, heart rate, and QOL in patients with coronary heart disease. The specific results may be different due to differences in individual conditions such as the age, gender, and course of the intervention samples. At the same time, the time, intensity, frequency, specific methods, and duration of the intervention will also affect the results. Therefore, the purpose of this meta-analysis is to evaluate the effects of mind-body exercise on the cardiopulmonary function, blood pressure, heart rate, and QOL in patients with coronary heart disease, and to provide specific exercise prescriptions about the time, intensity and frequency of mind-body exercise for patients with coronary heart disease.

## Author contributions

**Conceptualization:** Fei-xing Duan, Hui-xin Yang, Fei Dai, Lin Wang.

**Data curation:** Qi Wu, Yi-fan Zuo.

**Formal analysis:** Qi Wu, Yi-fan Zuo.

**Funding acquisition:** Qi Wu, Yi-fan Zuo, Lin Wang.

**Methodology:** Fei-xing Duan, Hui-xin Yang, Fei Dai.

**Resources:** Lin Wang.

**Software:** Fei-xing Duan, Fei Dai.

**Supervision:** Hui-xin Yang, Lin Wang.

**Validation:** Fei-xing Duan.

**Writing – original draft:** Fei-xing Duan.

**Writing – review & editing:** Hui-xin Yang, Lin Wang.
